# A rare case of massive myocardial infiltration by a disseminated extra‐nodal NK/T‐Cell lymphoma

**DOI:** 10.1002/jha2.396

**Published:** 2022-02-10

**Authors:** Firas Bayoudh, Aurélie Jaspers, Marie‐Agnès Azerad, Joan Somja, Hugues Marechal, Yves Beguin

**Affiliations:** ^1^ Department of Hematology, Centre Hospitalier Universitaire de Liège University of Liège Liège Belgium; ^2^ Department of Anatomical Pathology, Centre Hospitalier Universitaire de Liège University of Liège Liège Belgium; ^3^ Department of Intensive Care, Centre Hospitalier Régional de la Citadelle University of Liège Liège Belgium

A 38‐year‐old man was diagnosed with disseminated extra‐nodal NK/T‐cell lymphoma (ENKTL) with nasopharyngeal lesions, pleural effusions, and pericardial lesions. He was treated by chemotherapy (SMILE) followed by intensification with high dose chemotherapy (BEAM) and autologous hematopoietic stem cell transplantation, leading to complete remission. Five years later, he presented with fever, night sweats, cough, and elevated Epstein‐Barr Virus levels in the blood. Then, over a few days, he developed fluctuating binocular diplopia and painless skin bullae on his limbs. Fluorodeoxyglucose‐positron emission tomography/computed tomography suspected a disseminated relapse of ENKTL (with hypermetabolic lesions in the nasopharynx, lungs, pleura, heart, optical nerve, skin, lymph nodes, muscles, spleen, digestive tract, peritoneum).

Treatment was delayed by the first lockdown in Belgium due to the coronavirus disease 2019 pandemic and the refusal of the patient to be hospitalized. The situation suddenly deteriorated with atrial flutter, arterial hypotension, and hypoxia, and the patient was admitted to the intensive care unit.

**FIGURE 1:**
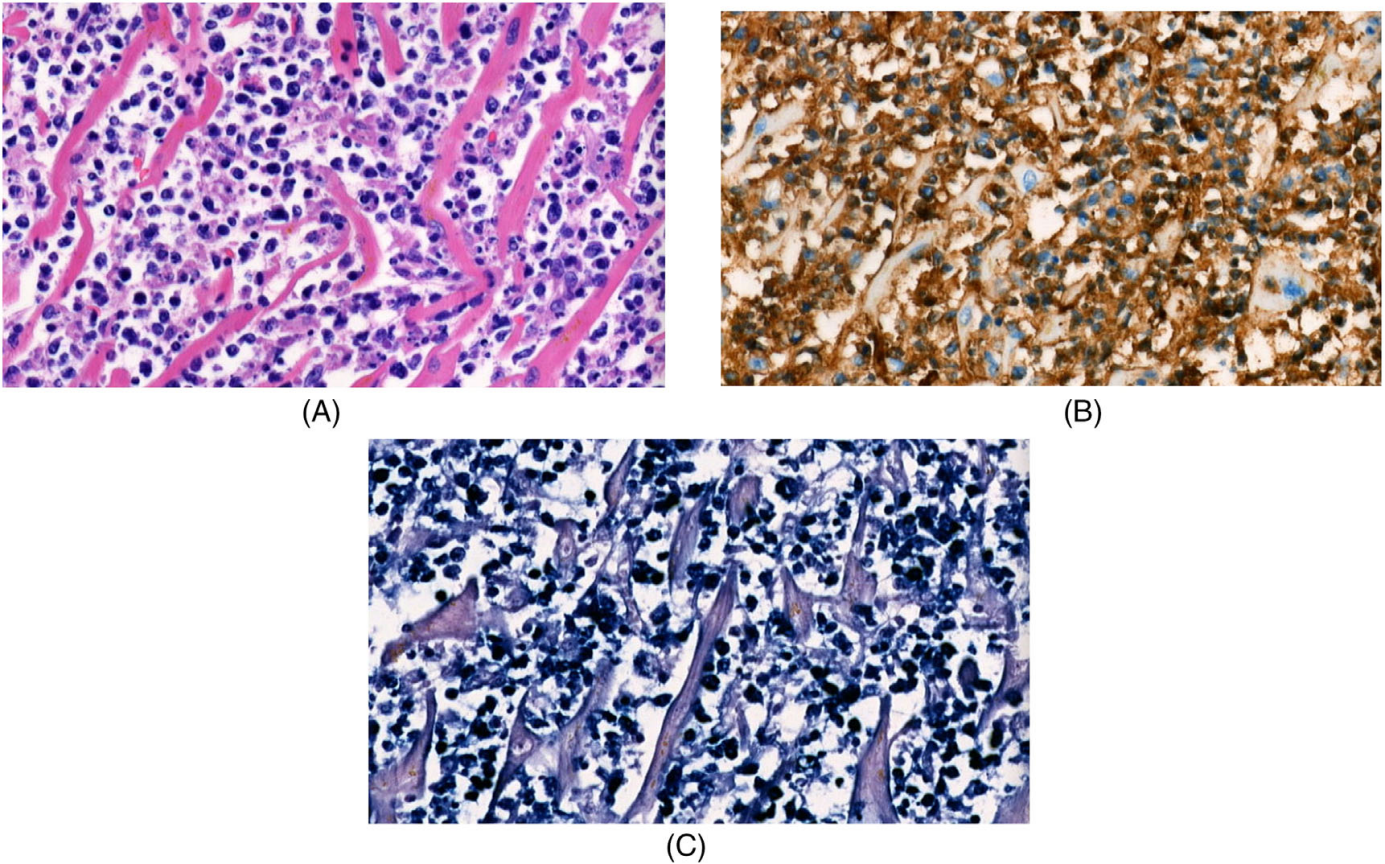
(A) Myocardial section (Hematoxylin and eosin) showing massive tumoral infiltration with small lymphocytes between myocytes. (B) Myocardial section confirms expressing of the natural killer cell marker CD56 on lymphocytes. (C) Myocardial section, presence of Epstein‐Barr Virus infected lymphocytes

Cardiac echography suspected tumoral infiltration of the interventricular septum with irregular images, myocardial hypertrophy, and pericardial effusion. Unfortunately, the patient died despite maximum supportive care. An autopsy confirmed multi‐organ infiltration by the ENKTL with massive myocardial invasion (images A, B, C) Figure 1. This is an atypical presentation for ENKTL, which is usually located in the aerodigestive tract when disseminated lesions are present [1]. The literature on extra‐nasal ENKTL is very limited with only a few case reports of myocardial infiltration [2, 3, 4, 5].
